# Multiple Mating But Not Recombination Causes Quantitative Increase in Offspring Genetic Diversity for Varying Genetic Architectures

**DOI:** 10.1371/journal.pone.0047220

**Published:** 2012-10-15

**Authors:** Olav Rueppell, Stephen Meier, Roland Deutsch

**Affiliations:** 1 Department of Biology, University of North Carolina, Greensboro, North Carolina, United States of America; 2 Department of Physics and Astronomy, University of North Carolina, Greensboro, North Carolina, United States of America; 3 Department of Mathematics and Statistics, University of North Carolina, Greensboro, North Carolina, United States of America; University of Lausanne, Switzerland

## Abstract

Explaining the evolution of sex and recombination is particularly intriguing for some species of eusocial insects because they display exceptionally high mating frequencies and genomic recombination rates. Explanations for both phenomena are based on the notion that both increase colony genetic diversity, with demonstrated benefits for colony disease resistance and division of labor. However, the relative contributions of mating number and recombination rate to colony genetic diversity have never been simultaneously assessed. Our study simulates colonies, assuming different mating numbers, recombination rates, and genetic architectures, to assess their worker genotypic diversity. The number of loci has a strong negative effect on genotypic diversity when the allelic effects are inversely scaled to locus number. In contrast, dominance, epistasis, lethal effects, or limiting the allelic diversity at each locus does not significantly affect the model outcomes. Mating number increases colony genotypic variance and lowers variation among colonies with quickly diminishing returns. Genomic recombination rate does not affect intra- and inter-colonial genotypic variance, regardless of mating frequency and genetic architecture. Recombination slightly increases the genotypic range of colonies and more strongly the number of workers with unique allele combinations across all loci. Overall, our study contradicts the argument that the exceptionally high recombination rates cause a quantitative increase in offspring genotypic diversity across one generation. Alternative explanations for the evolution of high recombination rates in social insects are therefore needed. Short-term benefits are central to most explanations of the evolution of multiple mating and high recombination rates in social insects but our results also apply to other species.

## Introduction

The evolution and the evolutionary maintenance of sex and genetic recombination continue to represent one of the central scientific problems of evolutionary biology. Theoretical explanations for the widespread occurrence of sex and recombination include short- and long-term benefits that are presumed to outweigh the fitness costs associated with these processes [1], [2]. Short-term benefits refer to the production of superior offspring, a process that is usually analyzed over one or a few generations [3], while long-term benefits are based on the recombination of genetic elements to form novel genotypes, a process that is usually studied over numerous generations [4]. While short-term benefits of sex and recombination can be interpreted as an increased mean and long-term benefits as an increased variance in a fitness-related trait [3], increased genetic variance may itself have direct, short-term benefits.

Variability and mean offspring fitness may relate to each other when competition among offspring is high [5]. While the idea that genetically diverse offspring may compete less with each other in a crowded environment (tangled bank hypothesis [6]) may not apply to the majority of species, it cannot be excluded in all cases [7]. A special case is provided by social insects, which are characterized by large numbers of philopatric offspring. In contrast to other taxa, the majority of these offspring represent non-reproductive workers that do not compete for direct reproduction. However, they are exploiting and living in the same environment and it has been predicted theoretically that high genetic diversity of workers increases colony performance by enhancing disease resistance [8], division of labor [9], and a number of other potential mechanisms [10], [11].

Experimental studies that compared genetically diverse colonies produced by multiply-mated queens to genetically more homogeneous colonies that were produced by single-mated queens of the Western Honey Bee (*Apis mellifera* L) demonstrated that genetically diverse colonies were better at resisting bacterial disease [12] and less prone to severe fungal infestations [13]. Similarly, high genetic diversity in experimental honey bee colonies improves their homeostasis [14], communication, foraging, and general colony success [15], [16]. Fitness benefits of increased colony genetic diversity by multiple mating have also been shown in other social insects, such as ants and bumblebees [17], [18].

Multiple mating by the female reproductives of social insect species increases the genetic diversity among their offspring and thus their colony [10], [19]. Concomitantly, multiple mating decreases inter-colony variance [20]. Although multiple mating among social insect females is considered relatively rare and evolutionarily derived [21], many species with large and complex societies have evolved multiple mating [22], with some female honey bees mating over 100 times [23]. Studies in many species have contributed to our understanding of the evolution of multiple mating in social insects. While no consensus has been reached, the influence of multiple mating on colony genetic diversity has emerged as the key factor for a general evolutionary explanation of multiple mating in social insects [22]. In addition to multiple mating, co-existing female reproductives also increase the genetic diversity in social insect colonies [24], but the primary selective reason for multiple co-existing females appears to be ecological limitations on independent colony founding [24], [25], [26].

The third process that has been invoked to increase colony genetic diversity is genetic recombination [27], [28]. All social insects studied so far show elevated recombination rates at the genome level [29], [30], [31]. Honey bees in particular exhibit the highest genome-wide recombination rates of all multicellular eukaryotes, with estimates exceeding 20cM/Mb [32] and a significant excess of recombination events per chromosome [29]. Despite some variability of the local recombination rate, *Apis mellifera* exhibits a high recombination across its entire genome, independently of chromosome size [29]. These results and the multiple independent evolution of high recombination in social insects [31] suggest that recombination patterns in social insects reflect a specific adaptive reason, rather than a general structural requirement for proper chromosome segregation. Similar to the explanation of multiple mating, several hypotheses exist to explain the high recombination rates in social insects [30], [32], [33], [34], including an alleged increase of colony genetic diversity by high recombination rates [27], [28].

Among social insect species, a negative relation between multiple mating and the occurrence of polygyny (existence of multiple functional female reproductives per colony) exists [24]. In contrast, the high recombination rates have been discovered in species that also exhibit very high queen mating numbers [28], [31], [32], [35]. This suggests that multiple mating and high recombination rates do not substitute for each other. Nevertheless, hypotheses that are based on the benefits of increased colony genetic diversity dominate the evolutionary explanation of both, multiple mating [11], [27] and high recombination rates [29], [30], [32], [36]. In contrast to mating, recombination rates are difficult to manipulate experimentally. Thus, no empirical studies on the effect of varying recombination have been performed in social insects.

The increase of colony genetic diversity by mating frequency and recombination rate has never been systematically evaluated. In this study, we simulated the relative impacts of multiple mating and recombination rate on the genotypic diversity of worker offspring of a single social insect queen with respect to an arbitrary, fitness-related trait. The majority of these traits have a complex genetic architecture [37], [38], [39], [40] and the genetic architecture of traits can have profound consequences for evolutionary outcomes [1], [41], [42]. For this reason, the simulations were performed with varying numbers of loci and models assuming different genetic architectures.

## Methods

### The Base Model

Based on the biology of the honey bee, the following simulation model was programmed and performed in “R”, version 2.12.0 [43]. One trait was assumed to influence colony performance and it was modeled to be influenced by *L* loci. These loci were randomly distributed over 101 potential locations on each of sixteen chromosomes (genome locations). These 1616 total genome locations represent a strong underestimation of the size of the honey bee genome [44] but make the following computations feasible. In addition, this size limitation increases any potential effects of recombination because loci are nearer to each other, increasing genetic linkage. For each locus, all parental alleles were initiated separately: two for the diploid mother queen and one for each of her haploid male mates (fathers). Allelic values were randomly and independently drawn from a standard normal distribution (mean = 0, standard deviation = 1). The queen was represented by a 2×*L* matrix of allelic values and each of her mates was represented by a 1×*L* matrix.

The model involved only one generation: The genotypes of 2000 worker offspring were generated from the parental genotypes according to the following rules of inheritance. For simplicity, all males had an equal probability of fathering offspring (but see [45]), and paternal loci were inherited as one completely-linked haplotype due to male haploidy. Thus, each worker was assigned the complete scalar of allelic values of its father. Maternal inheritance involved meiosis and recombination of the diploid maternal genome. The maternal allele of the first locus on each chromosome was selected at random from the two possible alternatives. The inheritance of all subsequent loci was then determined based on genetic linkage. For each of the 100 intervals per chromosome between adjacent genome locations, recombination was modeled to occur at the recombination rate *R*. A recombination event led to a phase shift and the inheritance of the alternative alleles along the chromosome until the next recombination (crossover). Double-crossovers were not allowed to occur within any one interval between genome locations. Crossover probability was not influenced by nearby crossover events, assuming no crossover interference [46] between adjacent intervals.

Any generated worker offspring was represented by a 2×*L* matrix of allelic values, with individual alleles inherited from its parents. The base model addressed only additive genetic effects. Therefore, once a worker's matrix was complete, the two allelic values for each locus were summed and the resulting locus values were averaged across all loci to calculate a worker's genotypic value. The contribution of all loci was weighted equally. Mutations that would change allelic values between parents and offspring were excluded from the model. The annotated “R” code of the base model is available from the Dryad web site (http://datadryad.org/; doi:10.5061/dryad.j57k3) and as electronic supplement (Text S1).

### Model Evaluation

The model was parameterized with twelve different numbers of mating partners per queen (M = 1, 2, 3, 4, 5, 7, 14, 21, 28, 35, 42, 49) that are biologically plausible for honey bees [47], [48], and a wide, log-linear set of recombination rates between adjacent loci (*R* = 0.0003125, 0.000625, 0.00125, 0.0025, 0.005, 0.01, 0.02, 0.04, 0.08, 0.16, 0.32, 0.64). With 101 loci per chromosome, these rates center on values of recombination events per chromosome that are typical for most animal species [29]. The number of influential loci was also varied over a wide range (*L* = 2, 4, 6, 8, 10, 14, 28, 42, 56, 70, 84, 98), adjusting the effect size of each locus by dividing its genotypic value by *L*. The model was also evaluated without the latter assumption (see below and Figure S1). The parameterization resulted in a total of 1728 (12×12×12) unique parameter combinations. Each of these simulation scenarios, generating worker offspring from parents across one generation, was evaluated 5000 times.

For each individual simulation, the genotypic value of 2000 workers was computed as a single number. The following summary statistics of these 2000 values were computed: the sample mean, the sample range, the sample variance, and the 95% confidence interval of the sample variance. Over all repeats we calculated the mean of the colony mean genotype values, the mean of the colony range of genotype values, the mean of the colony variance in genotype values, and the mean of the lower and upper limit of the 95% confidence interval of colony variance, as well as the variance of the colony means of genotypic values. The annotated “R” code for the simulation is also available from the Dryad web site (http://datadryad.org/; doi:10.5061/dryad.j57k3) and as electronic supplement (Text S1). Table 1 summarizes the assumptions for the model together with their biological justification and possible effects on the results.

**Table 1:** Assumptions of the model with their biological justification and possible effects.

Assumption	Justification	Possible Effects
No new mutations	Model simulates only one generation	None
Loci limited to 1616 genome positions	Computational simplification	Overestimation of recombination effect
Identical chromosomes	Conceptual simplification, chromosome size was evaluated in later model variation	Decreased model stochasticity
No paternity skew	Conceptual simplification	Decreased model stochasticity; overestimation of mating effect
No double-crossovers within the same genome interval	Probability low and empirical data [59]	Overestimation of recombination effect
No cross-over interference between adjacent genome intervals	Accurate computation not possible	Overestimation of recombination effect
Colony size = 2000	Computational simplification, natural colony sizes of social insects show a wide range	Upper limit to possible number of unique genotypes

We also verified the simulations analytically by computing the theoretical average value for the sample variance, 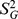, of the 2000 worker genotypic values in the simple case where recombination is not present. Assuming a trait influenced by *L* loci and a mating number *M*, the expected variance is given in Eq. 1. From there it can be seen that the variance increases linearly with the number of loci when locus effect is not scaled to locus number. The inverse is true when each locus effect size is scaled by 1/L. Also, the impact of the number of mates becomes relatively smaller as *M* increases.
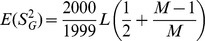



**Eq. 1:** *L*: Number of loci; M: Mating number

For a full analytical derivation of 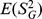 see the electronic supplement (Text S2).

### Model Variations

The following variations of the basic, additive model described above were evaluated. First, we relaxed the assumption that all loci contributed equally by drawing a specific weighting for each locus from a standard normal distribution. This weight vector was multiplied with the workers × loci matrix before workers genotypes were calculated. Another simple modification of the base model was to limit potential allelic values with equal probability to −1, 0, and 1. Alleles were randomly drawn from these three values instead of a normal distribution.

To evaluate a model including dominance effects, a dominance value ranging from 1–100 was randomly assigned to each allele. The genotypic value at each locus was then computed by multiplying each allele with its dominance value, adding these multiplication products, and dividing this sum by the sum of two dominance values. This model also included the variable weighting of loci. The dominance model was extended to include positive, negative, or neutral epistasis [42]. In all three cases, a loci × loci interaction matrix was drawn from a standard normal distribution, with the main diagonal elements set to zero to avoid epistasis of a locus with itself. For positive epistasis, each matrix value was increased by one, for negative epistasis one was subtracted, generating a mean epistasis coefficient of +1 and −1, respectively. The (directional) epistasis coefficient of each loci pair was then multiplied with the genotypic value of the respective epistatic locus to calculate the epistatic effect on the genotypic value of the other locus (for 2-locus example: Eq. 2). These modifications of the genotypic values for all loci were performed non-iteratively for each worker. The epistatic model was further extended by restricting allelic effects to −1, 0, and +1, instead of assuming an infinite number of alleles drawn from a normal distribution.
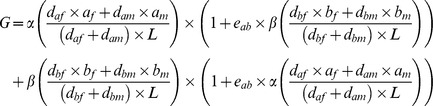



**Eq. 2:** G: Genotypic value; α,β: loci weights; d: allele-specific dominance values; a,b: allelic values; e: epistasis coefficients; *L*: Number of loci (in this case = 2)

Based on the finding that functionally equivalent alleles at the complimentary sex determination gene are lethal in honey bees [49], a further variation of the additive model was tested that assumed lethality at every locus if the allelic values were within 10% of each other. The evaluation of this model also included colony size as a dependent variable. To account for the possibility that the diversity of unique allele combination in a colony may be relevant, a qualitative model was constructed to assess the effect of multiple mating, recombination, and loci number on the number of unique allele combination in individuals of a colony. To generalize the model to different chromosome numbers [34], the epistasis model was evaluated for three loci numbers (*L = *10, 49, 98; mating numbers and recombination rates were parameterized as described above), assuming one, eight, sixteen, and sixty-four chromosomes, keeping the average number of recombination events across the entire genome constant.

### Statistical Analysis

Despite some significant departures from normality, the simulated data were evaluated with parametric test statistics throughout to consistently allow for regression analyses and calculation of partial correlation coefficients for evaluating the effect of the varied parameters within models. This is justified by the finding that for a given parameter set, colony values did not deviate significantly from parametric assumptions and by our large sample sizes [50]. In addition, we note that we perform statistical tests on the summary statistics of 5000 simulations, which could each constitute an independent data point. However, for computational and data management reasons, we did not store or analyze these raw data. Different model variations were compared with paired t-tests, controlling for the effect of *L*, *M*, and *R*. All statistical tests are meant to evaluate the relative importance of our variables and compare model variations because our data do not strictly represent empirical data, even though the individual simulations used stochastically drawn allele values.

### Ethics Statement

This study conforms to all applicable laws and regulations. It did not violate any ethical standards or require special permitting.

## Results

**Figure 1:**
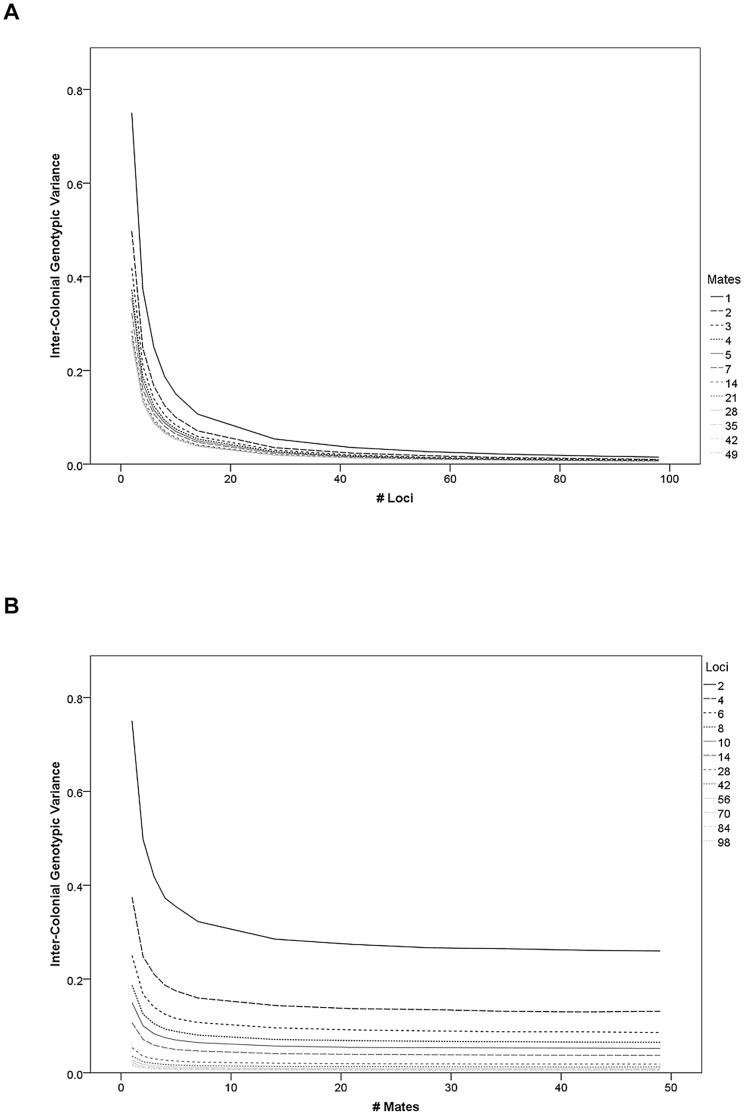
The inter-colonial variance of the mean genotypic worker values decreases with loci and mating number in the model. (a) The number of loci contributing to the genotypic variation strongly decreases the inter-colonial variance regardless of recombination rate. The effect is most pronounced when low mating frequencies are assumed. It decreases in strength with increasing locus number. (b) Mating frequency also decreases the inter-colonial variance of the mean genotypic values regardless of recombination rate, with quickly diminishing returns. The effect of mating frequency is most pronounced with few contributing loci.

The colony mean genotypic value across all tested parameter values was not significantly different from zero and did not correlate with *L*, *M*, or *R* in any of the models. In contrast, the inter-colonial variance of the colony mean was significantly reduced by *L* (partial correlation coefficient: r = −0.59, df = 1727, p<0.001) and *M* (r = −0.22, df = 1727, p<0.001) but not *R* (r = 0.00, df = 1727, p = 0.983). Inter-colonial variance exhibits additive, monotonous increases with *M* and *L*, but the effect of both factors is very weak for the upper half of the parameter space (Figure 1). Inter-colonial variance was not evaluated in the weighted, the dominance, and the qualitative model variations but all other variations (that also included weighting the loci differently and dominance effects) showed qualitatively equivalent results for the effect of *L*, *M*, and *R* on the inter-colonial variance of the average worker genotype (Table 2).

**Table 2:** Summary of the most important simulation results from the model and its variations (see main text for their explanation; significant effects in bold).

Model	Parameter	Effect of Mating number (*M*)	Effect of Recombination Rate (*R*)	Effect of Loci number (*L*)
Basic (Additive)	Inter-colonial variance	**r = −0.22, p<0.001**	r = 0.00, p = 0.983	**r = −0.59, p<0.001**
	Intra-colonial variance	**r = 0.14, p<0.001**	r = 0.00, p = 0.999	**r = −0.62, p<0.001**
	Range	**r = 0.51, p<0.001**	r = 0.02, p = 0.357	**r = −0.81, p<0.001**
3-Alleles Additive	Inter-colonial variance	**r = −0.22, p<0.001**	r = 0.00, p = 0.973	**r = −0.59, p<0.001**
	Intra-colonial variance	**r = 0.14, p<0.001**	r = 0.00, p = 0.999	**r = −0.62, p<0.001**
	Range	**r = 0.53, p<0.001**	r = 0.03, p = 0.278	**r = −0.83, p<0.001**
Weighted	Inter-colonial variance	n. det.	n. det.	n. det.
	Intra-colonial variance	**r = 0.14, p<0.001**	r = 0.00, p = 0.993	**r = −0.62, p<0.001**
	Range	**r = 0.57, p<0.001**	r = 0.02, p = 0.426	**r = −0.81, p<0.001**
Dominance	Inter-colonial variance	n. det.	n. det.	n. det.
	Intra-colonial variance	**r = 0.13, p<0.001**	r = 0.00, p = 0.999	**r = −0.62, p<0.001**
	Range	**r = 0.56, p<0.001**	r = 0.02, p = 0.413	**r = −0.80, p<0.001**
Neutral Epistasis	Inter-colonial variance	**r = −0.23, p<0.001**	r = 0.00, p = 0.984	**r = −0.58, p<0.001**
	Intra-colonial variance	**r = 0.13, p<0.001**	r = 0.00, p = 0.978	**r = −0.63, p<0.001**
	Range	**r = 0.56, p<0.001**	r = 0.02, p = 0.392	**r = −0.80, p<0.001**
Positive Epistasis	Inter-colonial variance	**r = −0.23, p<0.001**	r = 0.00, p = 0.977	**r = −0.58, p<0.001**
	Intra-colonial variance	**r = 0.13, p<0.001**	r = 0.00, p = 0.999	**r = −0.62, p<0.001**
	Range	**r = 0.56, p<0.001**	r = 0.02, p = 0.399	**r = −0.80, p<0.001**
Negative Epistasis	Inter-colonial variance	**r = −0.23, p<0.001**	r = 0.00, p = 0.935	**r = −0.58, p<0.001**
	Intra-colonial variance	**r = 0.13, p<0.001**	r = 0.00, p = 0.978	**r = −0.63, p<0.001**
	Range	**r = 0.56, p<0.001**	r = 0.02, p = 0.388	**r = −0.80, p<0.001**
3-Alleles Epistasis	Inter-colonial variance	**r = −0.23, p<0.001**	r = 0.00, p = 0.978	**r = −0.58, p<0.001**
	Intra-colonial variance	**r = 0.13, p<0.001**	r = 0.00, p = 0.998	**r = −0.62, p<0.001**
	Range	**r = 0.56, p<0.001**	r = 0.01, p = 0.310	**r = −0.84, p<0.001**
Lethality	Inter-colonial variance	**r = −0.23, p<0.001**	r = 0.00, p = 0.999	**r = −0.58, p<0.001**
	Intra-colonial variance	**r = 0.14, p<0.001**	r = 0.00, p = 0.990	**r = −0.62, p<0.001**
	Range	**r = 0.51, p<0.001**	r = 0.01, p = 0.349	**r = −0.82, p<0.001**
	Colony size	r = 0.00, p = 0.981	r = 0.00, p = 0.994	**r = −0.98, p<0.001**
Qualitative	# of unique genotypes	**r = 0.25, p<0.001**	**r = 0.05, p = 0.037**	**r = 0.68, p<0.001**

Comparing the effect of genetic architecture beyond loci number showed that restricting allele effects to three potential values for all loci decreased the inter-colonial variance relative to the unlimited number of potential allele effects under additive and epistatic conditions (paired t-test over all model parameterizations, respectively: t_(1727)_ = 29.9, p<0.001; t_(1727)_ = 29.2, p<0.001). Due to differences in the computational details, epistatic models could not be compared with additive models. Among the epistatic models with randomly drawn allelic values, variance was highest assuming positive epistasis, followed by neutral and then negative epistasis, although only the difference between positive and negative epistasis was significant (positive-negative: t_(1727)_ = 3.2, p = 0.002; positive-neutral: t_(1727)_ = 1.5, p = 0.137; neutral-negative: t_(1727)_ = 1.8, p = 0.066). The lethality model also reduced variance among colonies significantly compared to the simple additive model (t_(1,1727)_ = 6.0, p <0.001). However, this effect was dependent on *L*: Lethality only lowered the variance of colony means significantly for fewer than 14 loci. For 28 and more loci, lethality actually significantly increased inter-colony variance.

**Figure 2:**
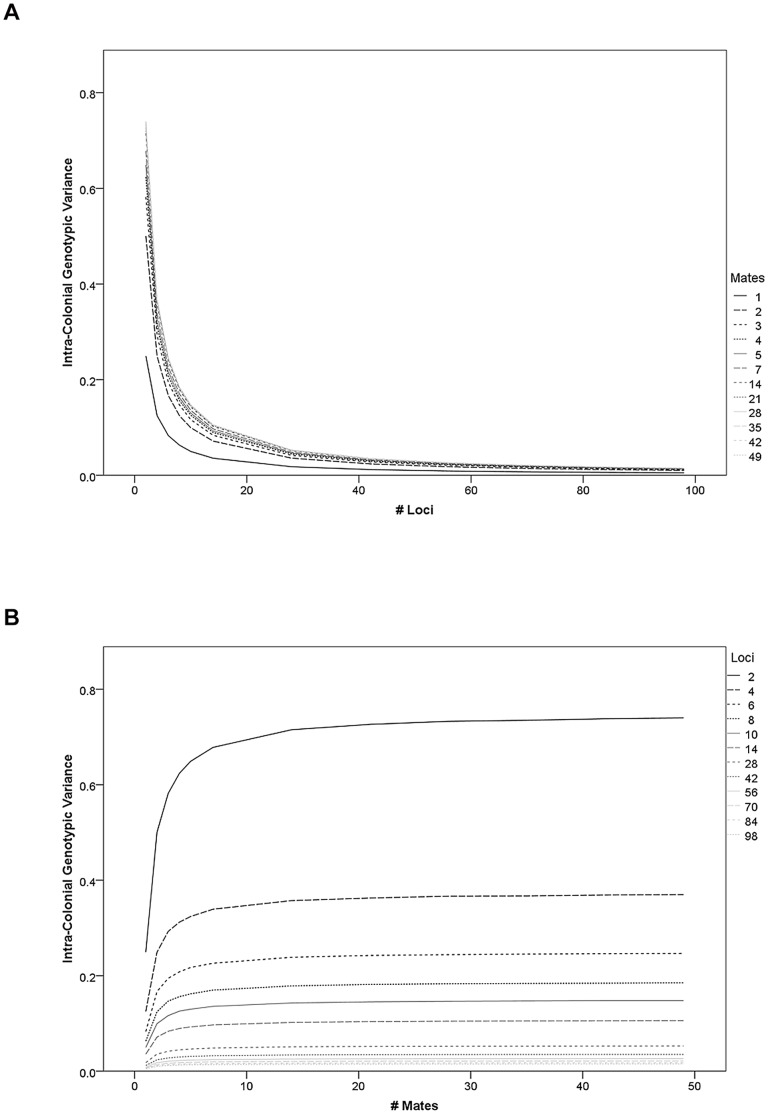
The intra-colonial variance of genotypic values of workers decreases with loci number but increases with mating number. (a) The intra-colonial variance decreases with increasing numbers of contributing loci. The effect is strongest for the initial increments of loci number and for high mating frequencies, but it is unaffected by genomic recombination rate. (b) Multiple mating increases intra-colonial genotypic variance regardless of recombination rates. The effect is most pronounced for *M*<10 and with few loci contributing.

The mean of the intra-colonial variance was also significantly influenced by *L* (r = −0.62, p<0.001) and *M* (r = 0.14, p<0.001) but not by *R* (r = 0.00, p = 0.999) in the basic model. The effect of L and M were again strongest in the lower part of the evaluated parameter space (Figure 2). All other models produced similar results for *L*, *R*, and *M* (Table 2).

The empirical estimate of the 95% confidence interval of the intra-colonial variance of genotypic values for each simulation scenario allowed a conservative estimate of significant differences between specific model scenarios: In the basic model, all scenarios with *L* = 2 had overlapping 95% CI. Similarly, all scenarios with 4, 6, 8, and 10 loci had overlapping 95% CI with all other scenarios of the same locus number. However, scenarios with low mating frequencies had significantly lower intra-colonial variance than scenarios with high mating frequencies, when *L*≥14. Conversely, *L* significantly decreased intra-colonial variance irrespective of *R* and *M*. Scenarios that only varied in *R* had overlapping 95% CI in all cases., The magnitude of the effects of *L* and *M* varied relative to the 95% CI in the different model variations, resulting in different degrees of overlap among model scenarios. However, all model variations agreed that scenarios that differed only with respect to *R* always had overlapping 95% CIs.

The intra-colonial genetic variance in the additive and epistatic models was significantly reduced by restricting the potential number of alleles to three (respectively: t_(1727)_ = 32.2, p<0.001; t_(1727)_ = 32.3, p<0.001). Weighing loci differently did not significantly affect the intra-colonial variance (t_(1727)_ = 1.2, p = 0.222), but lethality decreased intra-colonial variance (t_(1727)_ = 37.6, p<0.001). No meaningful comparisons of the additive model to dominance or epistasis models could be performed. While the epistatic model did not differ overall from the dominance model (t_(1727)_ = 0.0, p = 0.984), intra-colonial variance was higher in models of positive epistasis than in models of negative epistasis (t_(1727)_ = 4.6, p<0.001). Colony size in the lethality model was variable and significantly affected only by *L* (r = −0.98, p<0.001), not *M* (r = 0.00, p = 0.981) or *R* (r = 0.00, p = 0.994).

**Figure 3:**
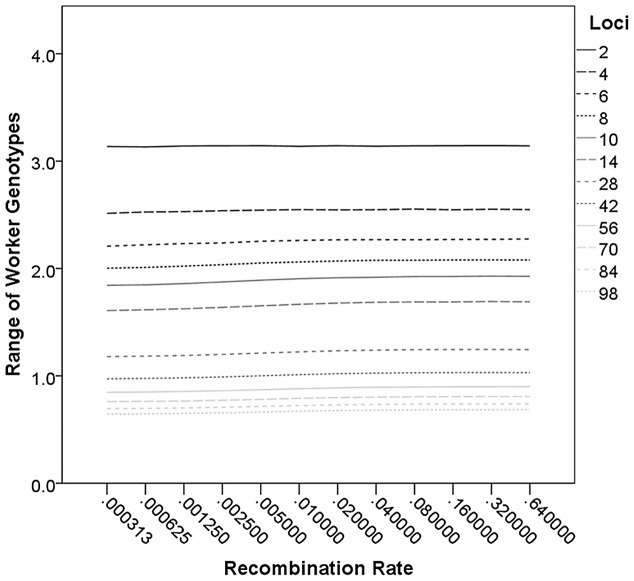
The range of worker genotype values in colonies is significantly affected by the number of contributing loci and queen mating frequency. However, the range also increases with increasing recombination rate. This effect is slight but most apparent for intermediate recombination rates when many loci contribute to the genotypic values.

In the basic model, the range of genotypic values of the workers in a colony was significantly correlated with *L* (r = −0.81, p<0.001) and *M* (r = 0.51, p<0.001) but not with *R* (r = 0.02, p = 0.357). All other models yielded similar results for the effect of *L* (r = −0.84–−0.80, p<0.001), *M* (r = 0.51–0.57, p<0.001), and *R* (r = 0.02–0.03, p>0.2). However, visual inspection of the effect of recombination on the range of genotypes revealed a slight positive effect of *R* in its intermediate parameter space when numerous loci were assumed (Figure 3). Therefore, the multiple regression of the genotypic range on L, M, and *R* was repeated for all models with more than one gene per chromosome on average (*L*>16). The results indicated a significant effect of *L* (r = −0.89, p<0.001), *M* (r = 0.76, p<0.001), and *R* (r = 0.12, p = 0.001). When the effects of *R* and M were assessed for each *L* independently, the partial correlation coefficient between *R* and the genotypic range increased monotonously from 0.00 (*L* = 2) to 0.16 (*L* = 98). Results from the analysis of the restricted data set (*L*>16) in all other models did not differ significantly with respect to the influence of *L* (r = −0.90–−0.86, p<0.001), *M* (r = 0.74–0.78, p<0.001), and *R* (r = 0.10–0.12, p = 0.001–0.003) on the genotypic range.

The genotypic range of colonies was also significantly decreased by restricting the potential allelic values to three (additive model: t_(1727)_ = 55.8, p<0.001; epistatic model: t_(1727)_ = 52.4, p<0.001). Positive epistasis significantly increased the range of genotypic values in a colony compared to neutral (t_(1727)_ = 10.0, p<0.001) and negative epistasis (t_(1727)_ = 11.5, p<0.001), with no significant difference between the latter two (t_(1727)_ = 0.9, p = 0.365). Lethality and weighting loci differently also decreased colony genotypic range overall (respectively: t_(1727)_ = 95.1, p<0.001; t_(1727)_ = 42.3, p<0.001), but there was no significant difference between the dominance and the basic epistasis model (t_(1727)_ = 0.5, p = 0.642).

**Figure 4:**
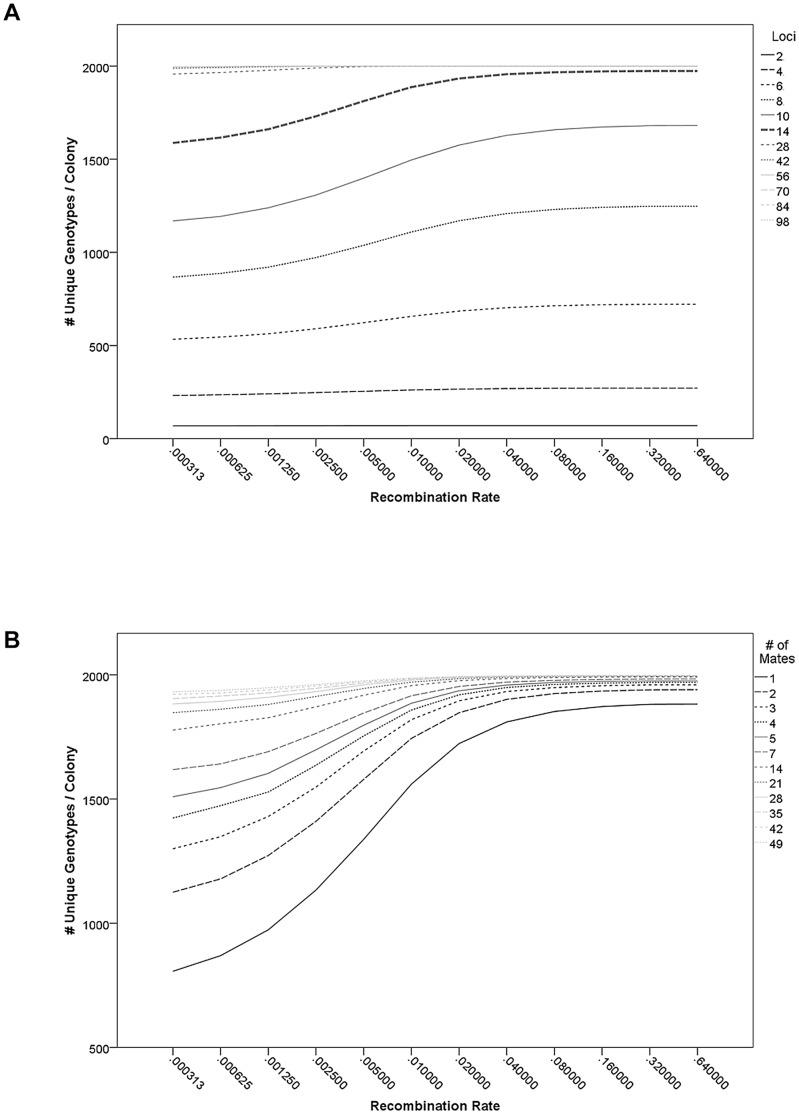
The number of unique allele combinations across all loci was strongly dependent on the number of contributing loci (a) and the number of matings by the queen (b). However, recombination rate also increased the number of unique genotypes in the lower portion of its parameter space and for intermediate numbers of contributing loci.

The number of unique allele combinations per colony irrespective of the genetic architecture was significantly influenced by *L* (r = 0.68, p<0.001), *M* (r = 0.25, p<0.001), and *R* (r = 0.05, p = 0.037). The effect of *R* on the number of unique allele combinations was strongest in scenarios with an intermediate number of loci (Figure 4). When the numbers of loci was low, the loci were mostly unlinked regardless of recombination rate. Conversely, high numbers of loci (*L*>42) resulted in almost all of the 2000 workers having unique allelic combinations, except for very small *R*. Regardless of *L* and *M*, the positive effect of *R* on the number of unique allele combinations per colony levels off between 0.04 and 0.08 (Figure 4).

**Figure 5:**
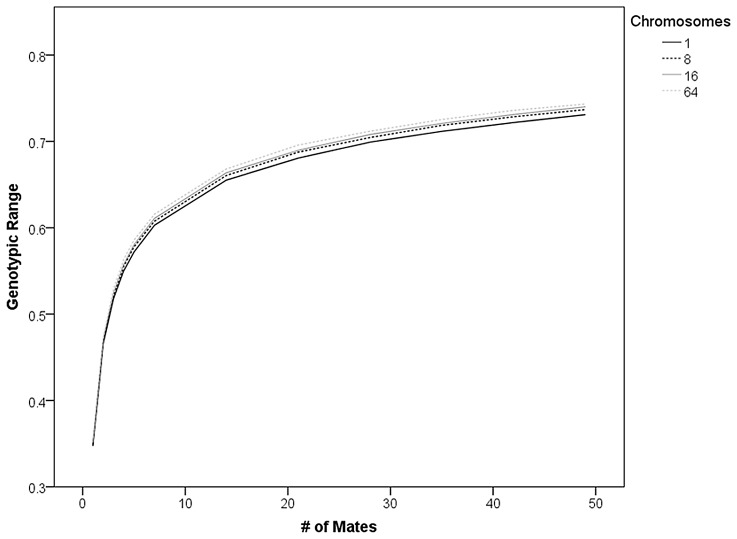
Higher chromosome numbers effectively increase the recombination rate. This effect increases the genotypic range but not intra- or inter-colonial genotypic variance across four different numbers of chromosomes when the effects of mating and loci number are statistically controlled for. The results of paired t-tests with Bonferroni correction are given.

Variation in chromosome number without changing overall genome size did not reveal any significant influences on the mean genotypic value, the intra- or inter-colonial variance of genotypic values, and the genotypic range within colonies that were not apparent from the basic model. Increasing the chromosome number increased the genotypic range but not the inter- and intra-colonial genotypic variance (Figure 5).

## Discussion

Some social insects have simultaneously evolved very high mating frequencies and genomic recombination rates. Motivated by these empirical observations, our simulations confirm previous studies that multiple mating increases the genetic diversity within colonies [10], [51] and lowers genetic variation among colonies [20]. However, the results show simultaneously that the genomic recombination rate does not influence the genotypic variance of quantitative traits in social insect colonies, contradicting the prevailing consensus in the literature [27], [28], [30], [32]. Thus, our study suggests that alternative explanations for the evolution of the exceptional recombination rates in social insects are needed. Increases in our third variable, the number of influential loci, consistently decreased intra- and inter-colonial genetic variance but did not affect the previous conclusions. Similarly, the results of models invoking more complex genetic architectures, such as epistatic interactions, differed little from the results of the basic additive model.

The recombination rate was varied across a wide range of possible values, from an average of 0.03 to 64 recombination events per chromosome. This range was centered on 1–2 recombination events per chromosome, which is close to empirical value of most species [52], [53]. However, varying the recombination rate did not significantly affect intra- or inter-colonial genetic variance. Relative changes in intra-colonial genetic variance due to recombination rate alone were smaller than 5% in all 144 different combinations of mating frequency and locus number in the additive model. Therefore, it seems unlikely that the high recombination rates of all advanced social insect species studied so far [28], [31], [32], [35], [54] have evolved because they quantitatively increased genetic diversity within colonies [30] or altered variance among colonies [20].

The results do not completely rule out genetic diversity hypotheses as explanation for the evolution of extreme recombination rates in the social insects. The range of genotypic values within colonies was slightly increased by elevated recombination rates in the empirically relevant, middle portion of the parameter space. It has been proposed before that uncommon phenotypic extremes may play an important role in colony function [51]. The relative changes in the genotypic range of colonies due to recombination rate alone in a given scenario of the basic model were ranging from 0.6% to 14.8%. The relative effect of recombination rate increased with locus number and decreased with multiple mating. Moreover, recombination rate exhibited a significant effect on the number of qualitatively unique genotypes per colony for intermediate numbers of loci and low mating frequencies. However, necessary evidence for multiple, qualitatively cooperating loci that affect colony efficiency is only beginning to emerge [38], [39], [55], [56], preventing a general assessment of their importance for colony fitness.

The genetic architecture of traits has profound consequences for their long-term evolutionary dynamics [41], [57]. Particularly, epistasis has been invoked in numerous models to explain evolutionary patterns, including the evolution of recombination [1], [42]. In contrast, dominance and epistasis effects did not have a marked influence on the outcomes of our model that only simulated short-term effects. Likewise, restricting the number of potential alleles per locus did not change the influence of recombination rate, queen mating frequency, and the number of contributing loci on any of the investigated response variables. The robustness of our results is due to the short-term nature of the model. We can conclude in general terms that the influence of mating and recombination on the quantitative genetic variance is not affected by the dominance, epistasis, or restricted allelic diversity over one generation. We note however, that the counting of qualitatively unique allele combinations represents a special case of strong epistasis, and that this model variation demonstrated a positive association between recombination and the number of unique allele combinations.

Another qualitative locus that has been characterized in several social insects is the complementary sex determining locus: the combination of two functionally equivalent alleles leads to diploid males, a lethal condition [58]. This phenomenon has also been related to the evolution of multiple mating [10] and therefore a model incorporating lethality was evaluated. This model variation did not differ from the basic model in any principle result. It generalized previous findings that the average colony fitness, quantified here as colony size, was unaffected by queen mating number in the presence of lethal loci [10]. Similarly, recombination rate had no effect on the average colony size, but the number of loci with lethal effects predictably decreased colony size.

The independent assortment of chromosomes during meiosis leads to recombination [33] and the relatively high chromosome number of honey bees (n = 16) might be decreasing the effect of intra-chromosomal recombination rates. However, the model variation that assessed different chromosome numbers did not exhibit an effect of recombination on the genetic variance measures and confirmed the effects of mating and locus number. However, it demonstrated that higher chromosome numbers lead to an increased genotypic range among the queen offspring, in accordance with the effect of recombination rate on the genotypic range in the other model variations.

Our results on the strong effect of multiple mating on intra- and inter-colonial genotypic variance conforms well with previous theoretical work [51], computer simulations of colony task performance [9], and empirical studies showing benefits of multiple mating for behavioral organization [14], disease resistance [17], and colony performance [16]. In concordance with previous studies the main benefit of multiple mating for genetic diversity is gained when mating numbers are low and the increase in variance becomes marginal for mating number above 20, irrespective of the number of contributing loci or other modifications of the genetic architecture.

Although our main results were surprisingly robust, our model variations revealed the importance of the genetic architecture of traits for their evolutionary dynamics. The number of segregating loci that affect a trait strongly decreases the genetic variation of this trait within and between families if the loci effects are scaled to the number of contributing loci. In preliminary versions of our model, we also considered the case that the magnitude of loci effects was independent of the number of contributing loci, which leads to the opposite conclusion that the number of loci increases intra- and inter-colonial genetic variance. Which of the two scenarios is closer to reality is an open, empirical question. We selected our main model because loci effects that are scaled to the number of contributing loci seem more plausible for most traits that are constrained to certain values. Comparing the model variations that extended the genetic architecture to non-additive effects and restricted genetic diversity in the general population also revealed interesting, one-generational consequences for the genotypic variability among individuals, suggesting that the ongoing empirical studies of genetic architecture have significant importance for understanding the evolutionary dynamics of complex traits.

## Supporting Information

Figure S1 **Graphical representation of the base model results when allelic effects are not scaled to the number of loci in the model.** This model variation changed the absolute values of the calculated variance and inverted the relationship between locus number and colony genotypic variance to a linear positive correlation. However, it did not affect conclusions about the effects of mating and recombination.
(PDF)

Text S1 **“R” code to simulate the worker genotypic values in a colony of social insects and the “R” code that was used in this study to batch-process the complete analysis of the base model.**
(DOC)

Text S2 **Shows the analytical derivation of the expected intra-colonial variance for the simple case of no genetic linkage between loci (R = 0.5).**
(PDF)
