# Supplementary material for: Multiple Mating But Not Recombination Causes Quantitative Increase in Offspring Genetic Diversity for Varying Genetic Architectures
Source: PLoS One. 2012 Oct 15;7(10):e47220. doi: 10.1371/journal.pone.0047220 (PMC3471945; doi:10.1371/journal.pone.0047220)
Supplement: Figure S1 — Graphical representation of the base model results when allelic effects are not scaled to the number of loci in the model. This model variation changed the absolute values of the calculated variance and inverted the relationship between locus number and colony genotypic variance to a linear positive correlation. However, it did not affect conclusions about the effects of mating and recombination. (PDF) [file pone.0047220.s001.pdf]

S2: Base model results when genotypic effects of loci are independent of (not scaled to) the number of loci that determine a trait. In this case, intra-colonial genetic variance increases linearly with locus number, but the positive effect of increased mating and the lack of effect of recombination are not significantly affected by this model modification.

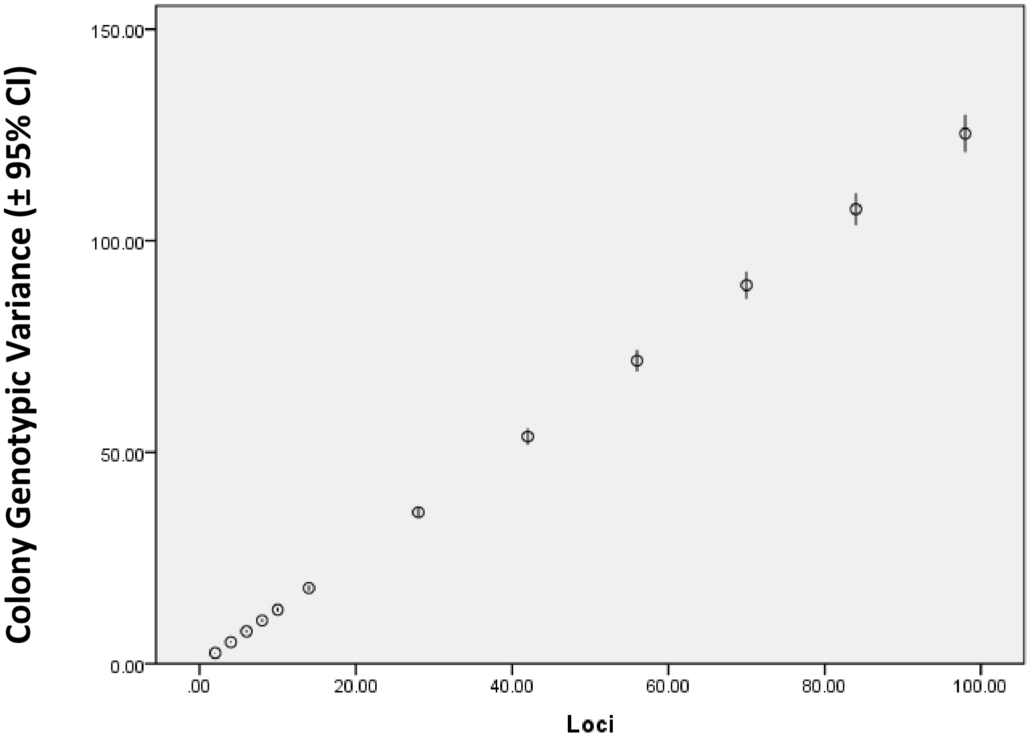

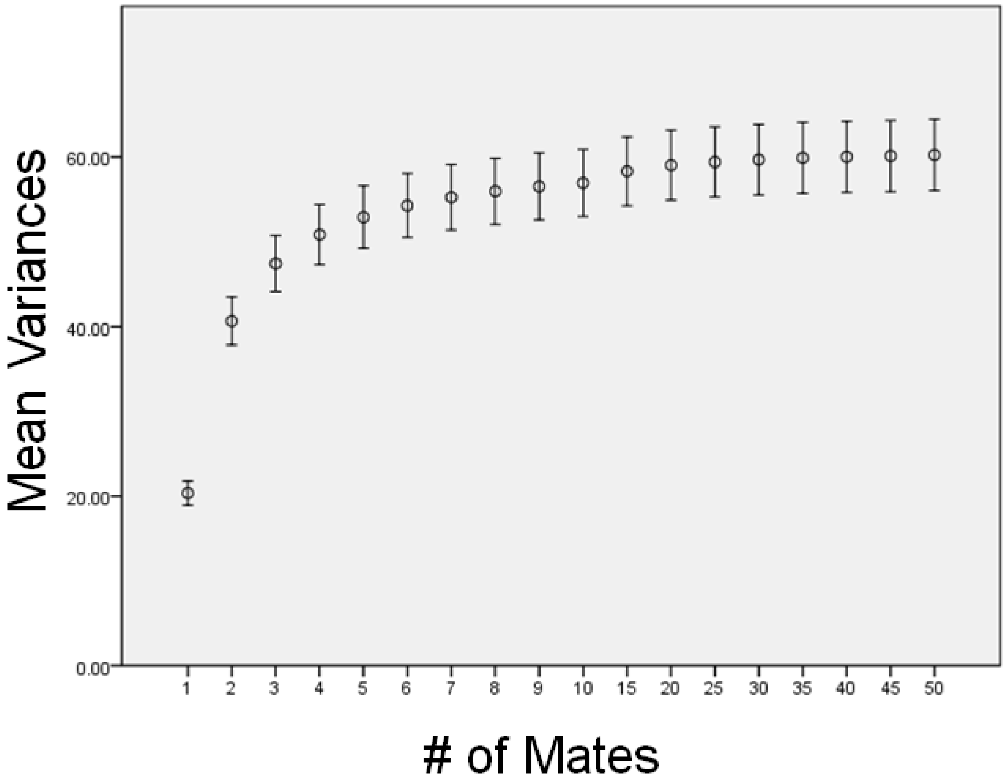

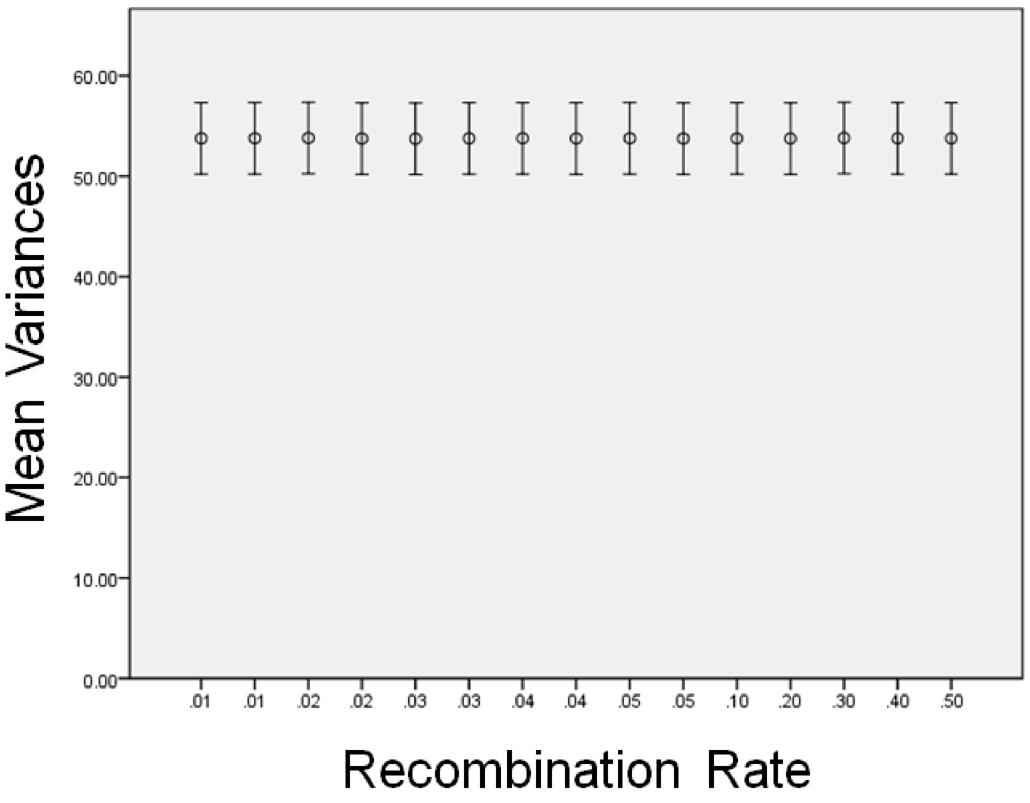

Mean Variances
60.00
50.00
40.00
30.00
20.00
10.00
0.00
.01
.01
.02
.02
.03
.03
.04
.04
.05
.05
.10
.20
.30
.40
.50
Recombination Rate
