# Supplementary material for: Multiple Mating But Not Recombination Causes Quantitative Increase in Offspring Genetic Diversity for Varying Genetic Architectures
Source: PLoS One. 2012 Oct 15;7(10):e47220. doi: 10.1371/journal.pone.0047220 (PMC3471945; doi:10.1371/journal.pone.0047220)
Supplement: Text S1 — “R” code to simulate the worker genotypic values in a colony of social insects and the “R” code that was used in this study to batch-process the complete analysis of the base model. (DOC) [file pone.0047220.s002.doc]

# “R” programming routines

Colony <- function(N.workers,N.mates,N.loci,R.rr){ # colony calculation procedure

Mates=matrix(rnorm(1*N.loci*N.mates),nrow=N.mates,ncol=N.loci) # mate matrix (mates by loci)

Queen=matrix(rnorm(2*N.loci*1),nrow=2,ncol=N.loci) # queen matrix (allele by loci)

Worker.Q=NULL # empty Worker.Q (matrix of queen alleles)

q.position=rep(0,N.loci) # allelic positions along chromosome Vector of length N.loci filled with "0"

q.chromosome=sort(sample(1:16,N.loci,replace=TRUE)) # random sampling of chromosomes (N.loci times) and sorting in increasing order to make vector

chrom.assigned=unique(q.chromosome) # put the chromosomes which contain loci in a vector

for(j in chrom.assigned){ # j will assume values of chromosomes that exist. i.e.: 1,5,9,15

freq.chrom=sum(q.chromosome==j) # how often the particular chromosome occurs

pos.number=sort(sample(1:101,freq.chrom,replace=FALSE)) # draw (freq.chrom) random position without replacement for j chromosome and sort them

q.position[q.chromosome==j]=pos.number # expanding q.position into a matrix (with chrom.assigned columns) and assign position numbers

}

select.allele=sample(1:2,N.workers,replace=TRUE) # makes a vector for all workers' starting phase of queen genotype

Worker.Q=cbind(Worker.Q,Queen[select.allele,1]) # c reates a vector of the first queen allele for each locus

if(N.loci>1){ # is true for all model parameterizations

for(i in 2:N.loci){ # attachment of the queen alleles for the remaining loci

if(q.chromosome[i]>q.chromosome[i-1]){ # if the next locus is on the next chromosome

select.allele=sample(1:2,N.workers,replace=TRUE) # random determination of the phase of the next chromosome

Worker.Q=cbind(Worker.Q,Queen[select.allele,i]) # add a column with randomly picked queen alleles at the ith locus

}

else{ # some genetic linkage because loci are on the same chromosome

dis=q.position[i]-q.position[i-1] # calculating the distance between the two loci

N.breaks=rbinom(N.workers,dis,R.rr) # calculating the number of breaks to the next locus

odd=((N.breaks%%2)>0) # determines whether an odd number of recombination events has occurred

prev.alleles = select.allele # passing on the phase of previous locus

select.allele[odd] = 3-prev.alleles[odd] # determine phase of current locus, selecting allele of correct phase

Worker.Q=cbind(Worker.Q,Queen[select.allele,i]) # adding allele to the worker matrix of queen alleles

}

}

}

select.dc=sample(1:N.mates,N.workers,replace=T) # randomly pick a drone contribution (a vector of drones)

Worker.D=Mates[select.dc,] # build up vector by picking length(select.dc) drone haplotypes

Worker=(Worker.Q/N.loci)+(Worker.D/N.loci) # add paternal and maternal contribution equally to each locus, scale by locus number

Gval= apply(Worker,1,sum) # calculate individual genotypic values

Gmean=mean(Gval) # calculate the mean of the colony

VGval=as.vector(var(Gval)) # calculate the variance of the colony

RGval=max(Gval)-min(Gval) # calculate the range of the colony

list(Geno.Var=VGval,Geno.Mean=Gmean, Geno.Range=RGval) # display the variance, mean, and range in a list that can be accessed

}

Simu <- function(N.Loci,N.Mates,Rec.Rate,N.sim=5000,Colony.Size=2000){ # main simulation procedure

l=length(N.Loci) # setting loop control variable

m=length(N.Mates) # setting loop control variable

r=length(Rec.Rate) # setting loop control variable

V.simulation=NULL # 3D-Matrix for mean variance results

V.of.V=NULL # 3D-Matrix for variance of variance results

Low=NULL # 3D-Matrix for lower boundary of 95%CI for mean variance results

High=NULL # 3D-Matrix for upper boundary of 95%CI for mean variance results

M.of.R=NULL # 3D-Matrix for mean range results

M.of.M=NULL # 3D-Matrix for mean of mean value results

V.of.M=NULL # 3D-Matrix for variance of mean results

for(i in 1:l){ # Loop to run simulation with different numbers of loci

for(j in 1:m){ # Loop to run simulation with different numbers of mates

for(k in 1:r){ # Loop to run simulation with different numbers of recombination rates

RVariances = NULL # Empty variables to accumulate results from subsequent loop (for o..)

RMeans=NULL

RRange=NULL

for(o in 1:N.sim){ # Loop to run simulation N.sim times (repeating the same scenario)

colo=Colony(Colony.Size,N.Mates[j],N.Loci[i],Rec.Rate[k]) # colo represents result vector with Geno.Var, Geno.Mean, Geno.Range

RVariances=c(RVariances,colo$Geno.Var) # Results accumulation in vector

RMeans=c(RMeans,colo$Geno.Mean)

RRange=c(RRange,colo$Geno.Range)

}

Means<-cumsum(RVariances)/(1:N.sim) # Vector that converges on the true mean of the colony variance (for initial assessment only)

V.simulation=c(V.simulation,i,j,k,mean(RVariances)) # Results matrices are gradually filled by concatenation

dim(V.simulation)<-c(4,k+(j-1)*r+(i-1)*m*r) # dimensions of data

Vnew.simulation=t(V.simulation) # transposition

V.of.V=c(V.of.V,i,j,k,var(RVariances)) # As above

dim(V.of.V)<-c(4,k+(j-1)*r+(i-1)*m*r)

Vnew.of.V=t(V.of.V)

Low=c(Low,i,j,k,quantile(RVariances,.025)) # As above

dim(Low)<-c(4,k+(j-1)*r+(i-1)*m*r)

Low.new=t(Low)

High=c(High,i,j,k,quantile(RVariances,.975)) # As above

dim(High)<-c(4,k+(j-1)*r+(i-1)*m*r)

High.new=t(High)

M.of.R=c(M.of.R,i,j,k,mean(RRange)) # As above

dim(M.of.R)<-c(4,k+(j-1)*r+(i-1)*m*r)

Range.new=t(M.of.R)

M.of.M=c(M.of.M,i,j,k,mean(RMeans)) # As above

dim(M.of.M)<-c(4,k+(j-1)*r+(i-1)*m*r)

Mean.new=t(M.of.M)

V.of.M=c(V.of.M,i,j,k,var(RMeans)) # As above

dim(V.of.M)<-c(4,k+(j-1)*r+(i-1)*m*r)

VarMean.new=t(V.of.M)

write.table(Vnew.simulation,file="M_V.txt",append=F) # Results are written into a permanent file

write.table(Vnew.of.V,file="V_V.txt",append=F)

write.table(Low.new,file="Lo_V.txt",append=F)

write.table(High.new,file="Hi_V.txt",append=F)

write.table(Mean.new,file="M_M.txt",append=F)

write.table(VarMean.new,file="V_M.txt",append=F)

write.table(Range.new,file="M_R.txt",append=F)

}

}

}

}

# Call of main simulation with evaluated parameter space:

Simu(c((1:5)*2,(1:7)*14),c(1:5,(1:7)*7),c(0.0003125,0.000625,0.00125,0.0025,0.005,0.01,0.02,0.04,0.08,0.16,0.32,0.64))
