# Supplementary material for: Multiple Mating But Not Recombination Causes Quantitative Increase in Offspring Genetic Diversity for Varying Genetic Architectures
Source: PLoS One. 2012 Oct 15;7(10):e47220. doi: 10.1371/journal.pone.0047220 (PMC3471945; doi:10.1371/journal.pone.0047220)
Supplement: Text S2 — Shows the analytical derivation of the expected intra-colonial variance for the simple case of no genetic linkage between loci (R = 0.5). (PDF) [file pone.0047220.s003.pdf]

1 Notation

To make the presentation for the derivation of the theoretical average value of S_G^2 more compact, we will use the following notation:

$X_i \stackrel{iid}{\sim} \mathbf{N}(0,1)$	$i = 1, \ldots, n$	The random variables $X_1, \ldots, X_n$ are independent of each other and all follow a standard normal distribution.
$Y \sim \mathbf{Bin}(n, p)$	....	The random variable Y has a binomial distribution based on n trials and a probability of success p.
$X \sim \boldsymbol{\chi}^2(df)$	....	The random variable X has a chi-squared distribution with df degrees of freedom.
$E(X)$	....	The (theoretical) average value of the random variable X.
$\mathrm{Var}(X)$	....	The (theoretical) variance of the random variable X.
$E(X\|Y)$	....	The (theoretical) average value of the random variable X given the value of the random variable Y.
$\mathrm{Var}(X\|Y)$	....	The (theoretical) variance of the random variable X given the value of the random variable Y.

2 Modeling a Colony of Worker Bees

In order to assess the variability of a colony based on the number of paternal lines, we are considering a simple model, where the genotypic values at each locus are standard normal random variables. In particular, when considering L loci and M paternal lines, the Queen of the colony is modeled as

$$Q = \begin{bmatrix} Q_1(1) & Q_1(2) & \ldots & Q_1(L) \\ Q_2(1) & Q_2(2) & \ldots & Q_2(L) \end{bmatrix}, \tag{1}$$

where $Q_a(l) \stackrel{iid}{\sim} \mathbf{N}(0,1),\ a = 1, 2,\ l = 1, \ldots, L$ represents the genotypic value on locus l and allele a of the Queen. Similarly, the M mates (drones) are modeled as

$$D = \begin{matrix} [D_1(1) & D_1(2) & \ldots & D_1(L)] \\ [D_2(1) & D_2(2) & \ldots & D_2(L)] \\ \vdots & \vdots & \ddots & \vdots \\ [D_M(1) & D_M(2) & \ldots & D_M(L)] \end{matrix}, \tag{2}$$

where $D_m(l) \overset{iid}{\sim} \mathbf{N}(0,1),\ m = 1,\ldots,M,\ l = 1,\ldots,L$ represents the genotypic value on locus l for mate m. With this, and disregarding recombination, any worker-offspring is modeled as follows: Each worker receives one allele from the Queen (without loss of generality we assume this to be the first allele) and one allele from one of the mates (we assume this to be the second allele). Since the mates are haploid, a mate will be selected as random to be the father of a specific worker and the worker inherits all genotypic values from that particular mate. As for the allele being inherited from the Queen, since she is diploid (as the workers) the worker could inherit either the genotypic value from the first or the second allele for any locus. Since we are also not considering recombination at the moment, all loci in the Queen can be seen as independent of each other, meaning that the inherited value on locus l has no influence on the inherited value at locus $l+1$. Formally, a worker can represented as

$$W = \begin{bmatrix} W_1(1) & W_1(2) & \ldots & W_1(L) \\ W_2(1) & W_2(2) & \ldots & W_2(L) \end{bmatrix} = \begin{bmatrix} Q_{a(1)}(1) & Q_{a(2)}(2) & \ldots & Q_{a(L)}(L) \\ D_m(1) & D_m(2) & \ldots & D_m(L) \end{bmatrix}, \quad (3)$$

where $a(l) = 1,2$ and $m = 1,\ldots,M$. Note that any of the $a(l) = 1$ with probability 0.5 and all values for m are equally likely with probability $\frac{1}{M}$.

3 Assessing Genotypic Variability of One Colony

Now, having a colony of n workers generated this way, we can compute the genetic variability of the colony by determining a genotypic value for each worker, G, and then compute the sample variance of all the G's. Given the model from above, the genotypic value of a single worker is computed as

$$G = \sum_{l=1}^{L}\big(Q_{a(l)}(l) + D_m(l)\big) = \sum_{l=1}^{L} Q_{a(l)}(l) + \sum_{l=1}^{L} D_m(l). \quad (4)$$

For the following, note that although the $Q_{a(l)}(l)$- and $D_m(l)$-values are generated from a standard normal distribution, this does not necessarily imply that the G-values themselves are normally distributed. In particular, for a given colony with a Queen and m mates, there are only finitely many values for G possible ($2^L M$ to be exact). Also, note that the $Q_{a(l)}(l)$-values are independent, whereas the $D_m(l)$-values are fully dependent (since the mates are haploid, specifying one $D_m(l)$-value automatically determines the other $L-1$

values). With these observations, the genotypic value of the ith worker can be rewritten as

$$G_i = \sum_{l=1}^{L} Q_{a_i(l)}(l) + \underbrace{\sum_{l=1}^{L} D_{m_i}(l)}_{=D_{m_i}} = Q_{a_i(1)}(1) + \ldots + Q_{a_i(L)}(L) + D_{m_i}. \tag{5}$$

The average of all the genotypic values of a colony is then given by

$$\begin{aligned}\bar{G} &= \frac{1}{n}\sum_{i=1}^{n} G_i = \frac{1}{n}\sum_{i=1}^{n} Q_{a_i(1)}(1) + \ldots + \frac{1}{n}\sum_{i=1}^{n} Q_{a_i(L)}(L) + \frac{1}{n}\sum_{i=1}^{n} D_{m_i} \\ &= \frac{1}{n}\sum_{l=1}^{L}\sum_{i=1}^{n} Q_{a_i(l)}(l) + \frac{1}{n}\sum_{i=1}^{n} D_{m_i} = \sum_{l=1}^{L} \bar{Q}(l) + \bar{D}.\end{aligned} \tag{6}$$

Now, since there are - as mentioned above - only finitely many genotypic values for the $Q_{a_i(l)}(l)$'s (2 at each locus) and the D_{m_i}'s (M total) the expression above can be written in their terms. Consider first $\bar{Q}(l)$: In a colony of n workers, $Y_1(l)$ workers will have $a(l) = 1$ and $n - Y_1(l)$ workers will have $a(l) = 2$. Hence,

$$\bar{Q}(l) = \frac{1}{n}\sum_{i=1}^{n} Q_{a_i(l)}(l) = \frac{1}{n}\Big(Y_1(l)Q_1(l) + \big(n - Y_1(l)\big)Q_2(l)\Big). \tag{7}$$

Note that $Y_1(l) \sim \mathbf{Bin}(n, 0.5)$ since both alleles have the same likelihood to be inherited to the worker. In similar fashion, we can rewrite $\bar{D}$. In a colony of n workers, Y_{21} will have the first mate as father, Y_{22} will have the second mate as father, etc. Clearly, $\sum_{m=1}^{M} Y_{2m} = n$. Since all mates have equal probability of being the father of a given worker, the random vector $Y_2 = (Y_{21}, \ldots, Y_{2M})'$ has a Multinomial distribution with parameters n and $\mathbf{m} = \left(\frac{1}{M}, \ldots, \frac{1}{M}\right)$. From this, we know that each Y_{2m} follows marginally a Binomial distribution, i.e. $Y_{2m} \sim \mathbf{Bin}\left(n, \frac{1}{M}\right)$. Thus,

$$\bar{D} = \frac{1}{n}\sum_{i=1}^{n} D_{m_i} = \frac{1}{n}\sum_{m=1}^{M} Y_{2m} D_m. \tag{8}$$

Having established the sample mean of the genotypic values of a colony, we can now assess the variability of the colony using the sample variance of the genotypic values:

$$
\begin{aligned}
S_G^2 &= \frac{1}{n-1}\sum_{i=1}^{n}(G_i-\bar{G})^2 = \frac{1}{n-1}\sum_{i=1}^{n}\left(\sum_{l=1}^{L} Q_{a_i(l)}(l) + D_{m_i} - \sum_{l=1}^{L}\bar{Q}(l) - \bar{D}\right)^2 \\
&= \frac{1}{n-1}\sum_{i=1}^{n}\left(\sum_{l=1}^{L}\big(Q_{a_i(l)}(l)-\bar{Q}(l)\big) + (D_{m_i}-\bar{D})\right)^2 \\
&= \sum_{l=1}^{L} S_{Q(l)}^2 + S_D^2 + 2\sum_{l=1}^{L-1}\sum_{k=l+1}^{L} S_{Q(l)Q(k)} + 2\sum_{l=1}^{L} S_{Q(l)D},
\end{aligned}
\tag{9}
$$

where

$$
\begin{aligned}
S_{Q(l)}^2 &= \frac{\sum_{i=1}^{n}\big(Q_{a_i(l)}(l)-\bar{Q}(l)\big)^2}{n-1} = \frac{Y_1(l)\big(Q_1(l)-\bar{Q}(l)\big)^2 + \big(n-Y_1(l)\big)\big(Q_2(l)-\bar{Q}(l)\big)^2}{n-1}, \\
S_D^2 &= \frac{\sum_{i=1}^{n}(D_{m_i}-\bar{D})^2}{n-1} = \frac{\sum_{m=1}^{L} Y_{2m}(D_m-\bar{D})^2}{n-1}, \\
S_{Q(l)Q(k)} &= \frac{1}{n-1}\sum_{i=1}^{n}\big(Q_{a_i(l)}(l)-\bar{Q}(l)\big)\big(Q_{a_i(k)}(k)-\bar{Q}(k)\big), \\
S_{Q(l)D} &= \frac{1}{n-1}\sum_{i=1}^{n}\big(Q_{a_i(l)}(l)-\bar{Q}(l)\big)\big(D_{m_i}-\bar{D}\big).
\end{aligned}
\tag{10}
$$

For large n, $S_{Q(l)Q(k)}$ and $S_{Q(l)D}$ will be close to 0 due to the aforementioned independence between the all $Q_{a(l)}(l)$- and D_m-values. This fact will be illustrated and used when computing the expectation of S_G^2. Also, note that the alternate expressions for $S_{Q(l)}^2$ and S_D^2 are derived by a similar argument as the alternate expressions for the means $\bar{Q}(l)$ and $\bar{D}$ and will also be useful in the computation of the expected value for S_G^2. Now, since the Y-variables are binomial random variables, we can take their expected values to compute the expected sample mean and expected sample variance for a colony of size n with fixed Queen and mates. Since $Y_1(l) \sim \mathbf{Bin}(n, 0.5)$, we know that $E\big(Y_1(l)\big) = \frac{n}{2}$. With this, we have

$$
E(\bar{Q}(l)|Q) = \frac{E\big(Y_1(l)\big)Q_1(l) + \Big(n - E\big(Y_1(l)\big)\Big)Q_2(l)}{n} = \frac{Q_1(l)+Q_2(l)}{2}. \tag{11}
$$

Also,

$$
\begin{aligned}
E(S_{Q(l)}^2|Q) &= \frac{E\big(Y_1(l)\big)\big(Q_1(l)-\bar{Q}(l)\big)^2 + \Big(n-E\big(Y_1(l)\big)\Big)\big(Q_2(l)-\bar{Q}(l)\big)^2}{n-1} \\
&= \frac{n}{n-1}\frac{1}{2}\sum_{a=1}^{2}\big(Q_a(l)-\bar{Q}(l)\big)^2.
\end{aligned}
\tag{12}
$$

In a similar fashion, we can find $E(\bar{D})$ and $E(S_D^2)$. We know that $Y_{2m} \sim \mathbf{Bin}\left(n, \frac{1}{M}\right)$ and thus $E(Y_{2m}) = \frac{n}{M}$. So,

$$E(\bar{D}|D) = \frac{1}{n}\sum_{m=1}^{M} E(Y_{2m})D_m = \frac{1}{M}\sum_{m=1}^{M} D_m \tag{13}$$

and

$$E(S_D^2|D) = \frac{\sum_{m=1}^{L} E(Y_{2m})(D_m - \bar{D})^2}{n-1} = \frac{n}{n{-}1}\frac{1}{M}\sum_{m=1}^{M}(D_m - \bar{D})^2. \tag{14}$$

We also could re-express $S_{Q(l)Q(k)}$ and $S_{Q(l)D}$ in similar manner, but we will forgo this here. With all that the expected sample variance of a colony with a specific Queen and a specific set of M mates can be written as

$$\begin{aligned} E(S_G^2|Q,D) = \frac{n}{n{-}1}&\left(\sum_{l=1}^{L}\frac{1}{2}\sum_{a=1}^{2}\big(Q_a(l) - \bar{Q}(l)\big)^2 + \frac{1}{M}\sum_{m=1}^{M}(D_m - \bar{D})^2\right) \\ &+ 2\left(\sum_{l=1}^{L-1}\sum_{k=l+1}^{L} S_{Q(l)Q(k)} + \sum_{l=1}^{L} S_{Q(l)D}\right). \end{aligned} \tag{15}$$

4 The Expected Value of Colony Variability

To compute the expected value of the sample variance of genotypic values over all possible colonies, we will use the fact that if $X_i \overset{iid}{\sim} \mathbf{N}(0, \sigma^2),\ i = 1, \ldots, n,$ then $\frac{\sum_{i=1}^{n}(X_i - \bar{X})^2}{\sigma^2} \sim \boldsymbol{\chi}^2(n{-}1)$ and thus $E\left(\frac{\sum_{i=1}^{n}(X_i - \bar{X})^2}{\sigma^2}\right) = n - 1$. Now, since $Q_a(l) \overset{iid}{\sim} \mathbf{N}(0,1),\ a = 1, 2$, it is easy to see that

$$\mathrm{Var}\big(Q(l)\big) = E\big(E(S_{Q(l)}^2|Q)\big) = \frac{n}{n{-}1}\frac{1}{2}. \tag{16}$$

To compute $\mathrm{Var}(D)$, note that $\sum_{l=1}^{L} D_m(l) \sim \mathbf{N}(0, L)$. With this $E\left(\frac{\sum_{m=1}^{M}(D_m - \bar{D})^2}{L}\right) = M - 1$ and hence

$$\mathrm{Var}(D) = E\big(E(S_D^2|D)\big) = \frac{n}{n-1}\frac{M-1}{M}L. \tag{17}$$

Now, due to the independence of $Q_{a(1)}(1), \ldots, Q_{a(M)}(M), D_m$, we know that $E\big(E(S_{Q(l)Q(k)}|Q)\big) = 0$ and $E\big(E(S_{Q(l)D}|Q,D)\big) = 0$. Thus,

$$\begin{aligned} E(S_G^2) = E\big(E(S_G^2|Q,D)\big) &= \sum_{l=1}^{L}\sigma_{Q(l)}^2 + \sigma_D^2 = \frac{n}{n{-}1}\frac{L}{2} + \frac{n}{n-1}\frac{M-1}{M}L \\ &= L\frac{n}{n{-}1}\left(\frac{1}{2} + \frac{M-1}{M}\right). \end{aligned} \tag{18}$$
